# Optimization of production of the anti-keratin 8 single-chain Fv TS1-218 in *Pichia pastoris *using design of experiments

**DOI:** 10.1186/1475-2859-10-34

**Published:** 2011-05-16

**Authors:** Rozbeh Jafari, Birgitta E Sundström, Patrik Holm

**Affiliations:** 1 Department of Chemistry and Biomedical Sciences, Karlstad University, S-651 88 Karlstad, Sweden

## Abstract

**Background:**

Optimization of conditions during recombinant protein production for improved yield is a major goal for protein scientists. Typically this is achieved by changing single crucial factor settings one at a time while other factors are kept fixed through trial-and-error experimentation. This approach may introduce larger bias and fail to identify interactions between the factors resulting in failure of finding the true optimal conditions.

**Results:**

In this study we have utilized design of experiments in order to identify optimal culture conditions with the aim to improve the final yield of the anti-keratin 8 scFv TS1-218, during expression in *P. pastoris *in shake flasks. The effect of: pH, temperature and methanol concentration on the yield of TS1-218 using buffered minimal medium was investigated and a predictive model established. The results demonstrated that higher starting pH and lower temperatures during induction significantly increased the yield of TS1-218. Furthermore, the result demonstrated increased biomass accumulation and cell viability at lower temperatures which suggested that the higher yield of TS1-218 could be attributed to lower protease activity in the culture medium. The optimal conditions (pH 7.1, temperature 11°C and methanol concentration 1.2%) suggested by the predictive model yielded 21.4 mg TS1-218 which is a 21-fold improvement compared to the yield prior to optimization.

**Conclusion:**

The results demonstrated that design of experiments can be utilized for a rapid optimization of initial culture conditions and that *P. pastoris *is highly capable of producing and secreting functional single-chain antibody fragments at temperatures as low as 11°C.

## Background

Single-chain variable fragments (scFv) are small recombinant antibodies that consist of the variable binding domains of the light and heavy-chain (V_L_, V_H_) joined together with a short peptide linker [1,2]. ScFvs retain the binding specificity of their parent immunoglobulins but are easier to manipulate and their expression is facilitated and can be readily expressed in different expression systems. During the last decades, the methylotropic yeast, *Pichia pastoris *(*P. pastoris*) has been proven to be a powerful candidate for high level expression of functional antibody fragments with reports of yields ranging from 10 mg up to 4.88 gram per liter of culture [3-9]. *P. pastoris *is easier to manipulate and culture than other eukaryotic cells and is also capable of performing many of the post-translational modifications seen in higher eukaryotes such as disulfide bond formation, glycosylation and proteolytic processing. In addition, *P. pastoris *provides the possibility of extracellular secretion of recombinant proteins and the low level of secreted endogenous *P. pastoris *proteins enables easier purification of recombinant proteins [10]. Recently, we have utilized the *P. pastoris *KM71H strain for overexpression of the anti-keratin 8 scFv (TS1-218) in shake flasks where a twenty-fold increase in yield of soluble TS1-218 was obtained compared to overexpression of the same scFv in *E. coli *[11]. However, the yield of TS1-218 from routine expression in *P. pastoris *using shake flasks could still become a substantial bottleneck for further progress due to the nature of shake flask cultures (i.e poor control).

Optimization of production conditions for overproduction of recombinant proteins are routinely achieved by varying single factors at a time until an apparent optimum is reached [7,8,12]. This approach could be labor intensive and assumes that all single parameters are mutually independent of one another and fails to identify interactions between the different factors involved [13]. The consequences could be failure in identifying the true optimal conditions for protein production. Recently, several groups have adopted the statistical design of experiments (DoE) methodology in order to address these limitations during optimization of the conditions in protein expression [14-18]. The major advantages of DoE are that interactions between multiple factors can be identified, and that a more reliable prediction of the true optimum can be achieved. In addition, a more structured approach towards experimental setup can be undertaken which can aid in reducing the number of experiments and facilitate data analysis. Today there are numerous software packages available which facilitate the application of DoE for non-statisticians.

In this study we have applied DoE for optimization of the initial culture conditions in order to improve the final yield of the TS1-218 during expression in shake flask cultures by the *P. pastoris *KM71H Mut^S ^strain. We have investigated whether yield of the TS1-218 can be improved without addition of supplementary compounds to the culture medium in order to facilitate downstream processing. Three factors; temperature, pH and methanol (MeOH) concentration and their effects, alone and in combination, were investigated using response surface methodology (RSM) [13]. Enzyme-Linked Immunosorbent Assay was used to assess the level of TS1-218 production.

## Methods

### Cell strains and media

*P. pastoris *strain KM71H Mut^S^; *arg4 aox1::ARG4 *(Invitrogen) expressing anti-keratin 8 scFv, TS1-218. The *P. pastoris *clone, was maintained on Yeast extract Peptone Dextrose medium (YPD) agar plates (1% (w/v) yeast extract, 2% (w/v) peptone, 2% (w/v) dextrose, 1% (w/v) agar). For biomass production, Buffered Minimal Glycerol medium (BMG; 100 mM potassium phosphate pH 6.0, 1.34% (w/v) yeast nitrogen base, 4 × 10^-5^% (w/v) biotin, 1% (v/v) glycerol) was used. Buffered Minimal Methanol medium (BMM; 100 mM potassium phosphate pH 4-8, 1.34% (w/v) yeast nitrogen base, 4 × 10^-5^% (w/v) biotin, 0.1-3% (v/v) MeOH was used for induction of protein expression.

### Experimental setup and analysis

Temperature and pH are two of the most common crucial factors that can influence protein production in various expression systems as well as production of scFvs in *P. pastoris *[7-9,19]. MeOH concentration is another important factor in protein expression in *P. pastoris *under the regulation of the alcohol oxidase promoter (AOX) since MeOH can be used as the inducer and sole carbon source [20,21]. Consequently these three factors; temperature, pH and MeOH concentration were chosen as quantitative factors in our investigation. The absorbance values from ELISA assays were chosen as a response for the level of TS1-218 production. In the final experiments the viability of the *P. pastoris *cells and change in wet cell weight (WCW) were also used as responses.

Response surface methodology (RSM) using Box-Behnken design [22] with three levels for each factor was employed to evaluate their effects and interactions on TS1-218 yield. In a Box-Behnken design the investigated points are located at the midpoints of the edges of a cubical design region and at the center. The Box-Behnken design offers an advantage when investigating three factors as fewer numbers of experimental runs are required compared to other RSM designs [13]. MODDE software version 7.0.0.1 (Umetrics AB, Sweden) was used for experimental setup and data analysis. A total of 12 experimental runs with different combinations of the three factors and four replicates of the center point were carried out (Table 1).

**Table 1:** Box-Benhken design matrix and experimental results for the different factors and their effect on the responses.

Experiment	pH	**Temp**°C	MeOH %	ABS	ΔWCW (%)	Viability %	pH_final_
N1	6	10	1.25	0.66	+4,1	99.73	5.2
N2	8	10	1.25	1.05	+2,2	99.85	6.5
N3	6	20	1.25	0.10	-5,3	99.02	5.1
N4	8	20	1.25	0.02	-1,0	99.34	6.7
N5	6	15	0.5	0.45	-5,2	99.56	4.5
N6	8	15	0.5	0.98	-1,5	99.37	6.6
N7	6	15	2.0	0.75	-4,4	99.62	5.0
N8	8	15	2.0	0.67	-1,8	99.56	6.6
N9	7	10	0.5	1.13	+3,5	99.45	6.2
N10	7	20	0.5	0.22	-5,0	99.02	6.2
N11	7	10	2.0	1.07	+2,2	99.67	6.3
N12	7	20	2.0	0.13	-5,2	99.16	6.4
N13*	7	15	1.25	1.16	-1,3	99.68	6.3
N14*	7	15	1.25	1.18	-1,1	99.74	6.2
N15*	7	15	1.25	1.12	-0,8	99.63	6.2
N16*	7	15	1.25	1.27	+1,2	99.66	6.2

*Centerpoint experiments

Temp: temperature (°C); MeOH: methanol concentration (%, v/v); ABS: absorbance at 405 nm with subtracted background (650 nm) reflecting TS1-218 yield; ΔWCW: change in wet cell weight (%) after 72 h of induction compared to WCW at start of induction (initial mean WCW: 564.4 ± SD 0.006 mg); pH_final_: pH of the culture medium after 72 h of induction.

### Expression of TS1-218

Single *P. pastoris *colonies were grown overnight in BMG medium at 30°C. The overnight culture was used to inoculate a larger volume of fresh BMG in baffled shake flasks (Nalgene). The cultures were grown until the cells reached log phase (OD_600 _2-6). The cultures were then divided equally between conical sterile 50 mL tubes and centrifuged at 1500 × g for 5 minutes at room temperature and the supernatant was discarded and the wet cell weights (WCW) were measured (mean WCW: 564.4 ± SD 0.006 mg). To induce protein expression the pellets were resuspended with 5 mL BMM to a final OD_600 _of ~50 and incubated for 72 h with vigorous shaking. Methanol was added (v/v) every 24 h to the cultures in order to maintain induction. The MeOH concentration and pH of the BMM medium and incubation temperatures varied depending on the experimental runs (Table 1). After 72 h of induction small samples were taken from the cultures for further analysis and the cultures were centrifuged at 3000 × g for 10 minutes at 4°C. The culture supernatants were stored at 4°C and the WCWs were measured.

### Cell viability assay

The viability of the *P. pastoris *cells were evaluated by propidium iodide (PI) staining [23]. Samples taken from the BMG cultures prior to induction and from each experimental run at the end point (72 h) of each experiment were diluted in phosphate buffered saline (PBS, 137 mM NaCl, 3 mM KCl, 8 mM Na_2_HPO_4 _and 1 mM KH_2_PO_4_, pH 7.4). The cells were stained with PI at a final concentration of 5 μg/mL for 10 minutes at room temperature. The samples were briefly centrifuged at 1500 × g and the supernatants were discarded. The cells were washed twice with PBS and finally analyzed in a BD FACScan™ flow cytometer (Becton Dickinson). A total count of 50000 cells was collected for each sample. PI negative cells were considered as viable cells and PI positive cells were regarded as dead. As a positive control, heat-killed *P. pastoris *cells were stained with PI as described above. As negative control, *P. pastoris *cells without PI staining were used.

### Enzyme Linked Immunosorbent Assay

Microtiter 96 wells plates (Nunc) were coated over night at 4°C with 100 μL per well of 5 μg/mL keratin 8/18/19 complex in 0.1 mM NaOH [24]. The culture medium from different runs where diluted 60 times with (50 mM sodium phosphate buffer pH 6.5, 350 mM NaCl) and added in triplicates with 100 μL sample per well. The plates were incubated on a shaker for 2 h at room temperature. After washing with 50 mM Tris-HCl pH 7.4, 150 mM NaCl and 0.1% (v/v) Tween-20 (TBST) the plates were incubated for 2 h on a shaker at room temperature with 100 μL per well (0.85 μg/mL) monoclonal mouse anti-polyhistidine-alkaline phosphatase conjugated antibody clone his-1 (Sigma-Aldrich) diluted with TBST. After washing with TBST the plates were developed with 3 mM para-Nitro Phenyl Phosphate (pNPP) in 50 mM 2-amino-2-metyl-1-propanol, 1 mM MgCl_2 _pH10. The absorbance was measured at 405 nm and the absorbance at 650 nm was subtracted. The mean absorbance for triplicate samples were calculated for the different runs before analysis in MODDE.

### Purification of TS1-218

The hexahistidine-tagged TS1-218 from the scaled up one liter cultures were purified from the culture medium with immobilized-metal affinity chromatography (IMAC) using Ni-NTA agarose (Invitrogen). The TS1-218 in the culture medium were allowed to bind to a 10 mL Ni-NTA agarose gel bed in a K26/60 column (Pharmacia) followed by a wash of the Ni-NTA agarose gel bed with wash buffer (50 mM sodium phosphate buffer pH 6.5, 350 mM NaCl) and another wash with a wash buffer containing 25 mM imidazole. The TS1-218 was eluted with 50 mM sodium phosphate buffer pH 6.5, 350 mM NaCl, 300 mM imidazole. The samples were concentrated and the buffer was exchanged against wash buffer using Amicon Ultra-15 centrifugal filter units. BCA Protein Assay Kit (Pierce) was used to determine the concentration of TS1-218.

### SDS-PAGE

The purified TS1-218 samples from the validations experiments were adjusted to equal volumes and separated on NuPage^® ^Novex Bis-Tris 4-12% polyacrylamide gels with NuPAGE^® ^MES SDS Running Buffer according to the manufacturer's recommendations (Invitrogen) and stained with Coomassie Blue R-250.

## Results and Discussion

The aim of this study was to identify optimal culture conditions for TS1-218 production in BMM medium in shake flasks by the methylotrophic yeast *P. pastoris *using design of experiments. There are many different factors that can influence protein expression in *P. pastoris*, such as pH, temperature, MeOH concentration, cell density, medium composition or additives (casamino acids, sorbitol, EDTA) to name a few. In a previous study, *P. pastoris *KM71H/Mut^S ^clones expressing hexahistidine-tagged (His-tagged) TS1-218 under the control of the AOX promoter were cultured in complex medium containing yeast extract and peptone at pH 6, temperature 20°C and 0.5% (v/v) methanol during expression [11]. Some components of the complex medium were retained in the metal affinity purification matrix and co-eluted with the TS1-218. These components interfered with the downstream processing of the purified proteins and needed to be removed prior to further analysis. Additives such as casamino acids and EDTA have been shown to increase the yield of secreted proteins in many cases [7,25,26], probably due to their inhibitory effect on extracellular proteases present in the culture medium. However, these medium additives can also interfere with the purification matrix during affinity purification of His-tagged proteins as mentioned earlier. For these reasons, buffered minimal medium without any supplementary additives which provides less background was used for both biomass production and protein induction in this study.

Generally, when using *P. pastoris *Mut^S ^strains for protein production, high cell densities are used since the yield of secreted proteins are proportional to cell density [27]. In addition, the Mut^S ^strains grow at a slower rate when methanol is used as the sole carbon source. However, very high cell densities can result in rapid depletion of the nutrients and lead to increased accumulation of extracellular protease activity due to increased cell death [20,27]. In contrast, in low cell density cultures more resources are employed for biomass production than protein production [7,8]. Therefore we decided to use a cell density (OD_600_) of ~50 during the induction phase throughout the entire study.

Initially the effect of pH, temperature and MeOH concentration were investigated using a wide range between the factor levels; pH 4-8, temperature 15-30°C and MeOH concentration 0.1-3%. However, at the highest temperature (30°C) and lowest pH (pH 4) investigated, no signal were detected in the ELISA assays for any of the experimental runs, nor were there any TS1-218 detected after Ni-NTA purification and SDS-PAGE analysis of the culture medium from these runs (results not shown). The results from the initial experiment indicated that lower temperatures and a more narrow range of the factors should be investigated in order to achieve a more comprehensive model.

**Figure 1:**
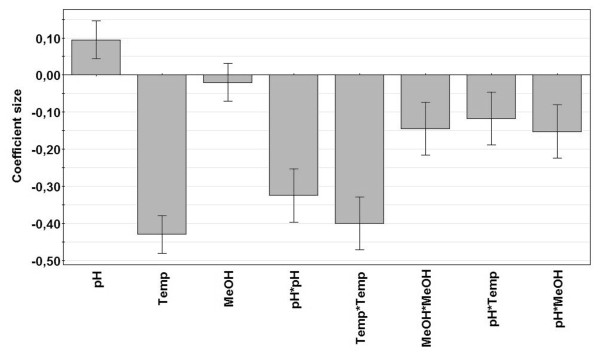
**Coefficient plot of the ELISA ABS response**. Coefficient plot with confidence intervals (error bars) showing the main (pH, Temp, MeOH concentration), squared (pH*pH, Temp*Temp, MeOH*MeOH) and interaction (pH*Temp, pH*MeOH) effects of the investigated factors on the ELISA absorbance response. The significance level was set at 95%. The significance of each term (factor) in the model is determined by the size of the coefficients (bars) and their error bars. Non-significant terms have an error bar that spans zero (p > 0.05). A negative coefficient for a main factor indicates that a decrease in that factor setting, relative to its center point, will have a positive effect on the yield of TS1-218 and consequently result in higher absorbance values. The interaction term temp*MeOH was not significant and was therefore removed from the model. The main term MeOH was also not significant, however, removing the term MeOH would also have removed the MeOH*MeOH and pH*MeOH terms which were significant.

In the second series of experiments, factor levels; pH 6-8, temperature 10-20°C and MeOH concentration of 0.5-2% were investigated in a Box-Behnken design (Table 1). The absorbance data were fitted with multiple linear regression (MLR) which showed a statistically good model with a model validity of 0.86 which demonstrated that there were no lack of fit (p > 0.05). Model validity larger than 0.25 indicates that the model error is in the same range as the pure error and a model validity of 1 represents a perfect model. The model indicated that 99% of the absorbance response variation could be explained by the model (R^2 ^= 0.99) and that 94% of the absorbance response variation could be predicted (Q^2 ^= 0.94). The reproducibility of the model (variation of the center point runs) was 0.98. A reproducibility of 1.0 indicates that the pure error is zero. The coefficient plot demonstrated that the main factors pH and temperature significantly influenced the yield of TS1-218 whereas different MeOH concentration did not influence the final TS1-218 yield (Figure 1). An interaction between pH and temperature as well as between pH and MeOH concentration could also be identified. Overall the model showed that the pH and temperature had the highest impact on TS1-218 yield as they presented the largest coefficients (Figure 1). The results obtained demonstrated that increasing the initial pH of the culture medium and decreasing temperature during induction resulted in higher absorbance values in the ELISA assays which reflect a higher TS1-218 concentration in the culture medium.

**Figure 2:**
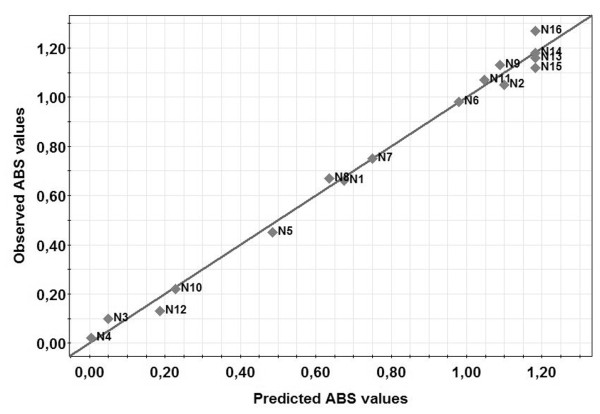
**Demonstration of the predictive ability of the model**. The scatter plot shows the predicted versus measured experimental absorbance values. The fit to the line of parity was shown with R^2 ^= 0.99 which indicated that the regression model was able to predict output data with high reliability.

**Figure 3:**
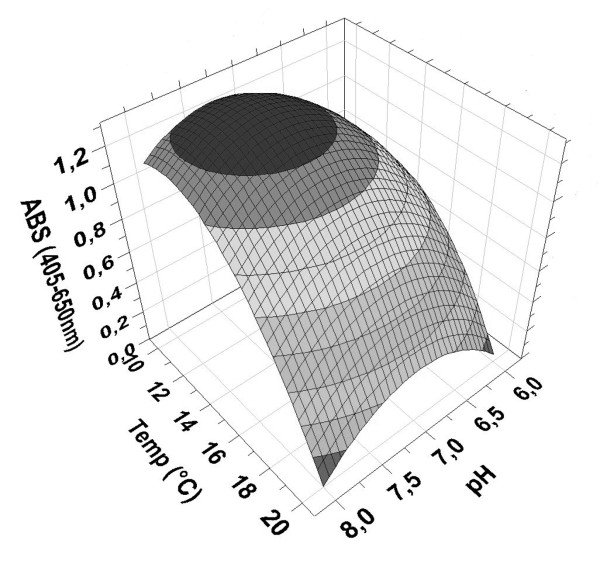
**Response surface plot of the ELISA ABS response**. Response surface plot showing the influence of temperature and pH on the yield of TS1-218 reflected by the absorbance signal from the ELISA. The MeOH concentration was at the center point level (1.25%).

**Figure 4:**
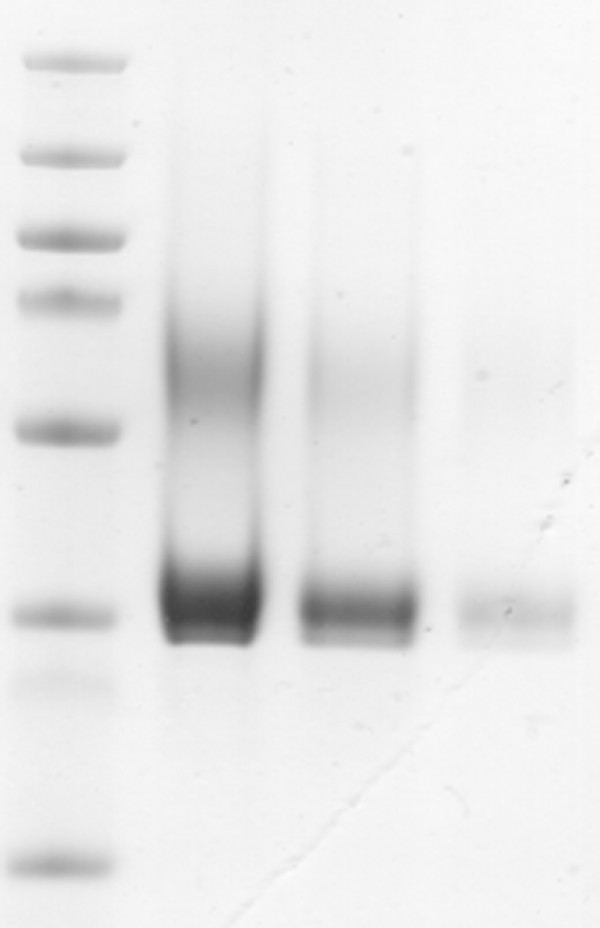
**Visualization of the increased scFv yield after optimization using SDS-PAGE**. Purified TS1-218 expressed under different culture conditions visualized by Coomassie Blue staining. Lane 1: Molecular weight marker PageRuler Plus (Fermentas); Lane 2: Optimized conditions (pH 7.1, 1.2% MeOH, 11°C); Lane 3: Reference conditions (pH 7.5, 1% MeOH, 15.5°C); Lane 4: Pre-optimization conditions (pH 6, 0.5% MeOH, 20°C).

The correlation between the predictive and measured values for the ELISA absorbance values demonstrated an excellent parity and predictive capacity (Figure 2). The response surface plot of the ELISA absorbance response clearly indicated that lower temperature and higher initial pH should be employed when attempting to increase the yield of TS1-218 (Figure 3). According to the model an optimum for maximizing TS1-218 yield could be reached using BMM medium with a pH of 7.1, a MeOH concentration of 1.2% and a temperature of 11°C during induction. To validate the predicted optimum for maximum yield of TS1-218, cultures were scaled up to 1 liter cultures in baffled shake flasks. This resulted in a yield of 21.4 ± 1.25 mg TS1-218 (n = 3) at the optimal factor settings after purification which can be compared to ~1 mg TS1-218 obtained prior to optimization (Table 2). In addition, for comparison the factor settings (pH 7.5, 1% MeOH concentration, temp 15.5°C) were investigated which yielded 15.3 ± 1.3 mg TS1-218 (n = 3) (Table 2). The observed results correlated with the predictions of the model regarding the TS1-218 production where an increased TS1-218 yield could be obtained. The samples from the validation experiments were analyzed using SDS-PAGE which demonstrated the higher yield after optimization of the culture conditions (Figure 4). The purity of the samples were in line with our previous study where a TS1-218 double band as well as a portion of glycosylated form of TS1-218 could be observed [11].

**Table 2:** Validation of the predicted optimal culture conditions for optimal TS1-218 yield in one liter cultures.

Experiment	pH	**Temp **°C	MeOH %	Yield (mg)
Pre-Optimization	6	20	0.5	1.0 ± 0.14
Reference	7.5	15.5	1	15.3 ± 1.29
Optimized	7.1	11	1.2	21.4 ± 1.25

Temp: temperature (°C); MeOH: methanol concentration (%, v/v). Results are derived from three different experimental runs. Statistical significant difference was observed between every experiment with regard to the final yields (p < 0.05).

*P. pastoris *has several endogenous proteases although very little is known about these different proteases. Shi et al. [7] have described an increased production of a scFv at higher pH (7.5-8) in shake flask cultures, although the scFv production did not correlate with protease activity. Other studies have however shown that lower pH in the bioreactor cultures resulted in increased protease activity, which suggest that the majority of the proteases released by *P. pastoris *in these studies have an optimal activity at acidic conditions [28,29]. When measuring the pH in the culture medium after 72 h of induction it was found that the pH had decreased markedly in the different experimental runs even though a buffered medium was used (Table 1). It is likely that the TS1-218 was also subjected to increased proteolytic degradation in culture medium with lower pH resulting in weaker absorbance signal in the ELISA assays for the experimental runs with a lower starting pH. This could probably be avoided if the pH in the cultures could be maintained at strict intervals by improving the buffer capacity in the culture medium or using bioreactors.

**Figure 5:**
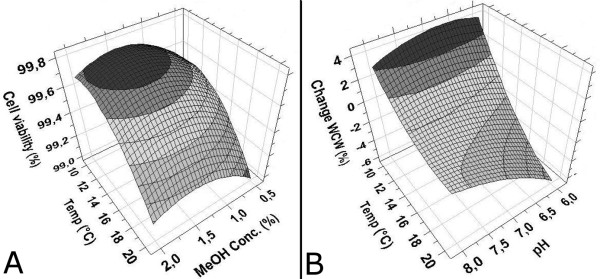
**Response surface plots of the cell viability and WCW responses**. Response surface plots of the optimization model showing the influence of temperature and MeOH concentration on the viability of the *P. pastoris *cells **(A) **and the effect of temperature and pH on the biomass (i.e. change of WCW in %) after 72 h of induction **(B)**. The pH did not have a significant effect on the viability of the *P. pastoris *cells. The MeOH concentration of the response surface plot in panel B was at the center point level (1.25%).

In a study by Dragosits et al. [30] no difference could be seen in the proteolytic activity in culture supernatants cultivated at different temperatures (20, 25 and 30°C). On the contrary Hong et al. [28] have reported a decreased proteolytic activity in samples taken from fermentations performed at lower temperatures. In another study, Li et al. [31] reported that their recombinant protein was degraded after two days when expressed at 30°C but not at 23°C. This was attributed to lower amounts of extracellular protease in the culture medium due to increased cell viability at lower temperatures. It is commonly believed that intracellular *P. pastoris *proteases are released extracellularly upon death of the *P. pastoris *cells. When analyzing the viability of the *P. pastoris *cells after 72 h of induction the results demonstrated a higher viability at lower temperatures compared to the higher temperatures (Figure 5A). Although the viability of the *P. pastoris *cells did not decrease below 99% for any of the experimental runs, temperature was shown to have a significant impact on the viability (p < 0.05). This was in line with the change in wet cell weight (WCW) which demonstrated a statistically significant increase (p < 0.05) at lower temperatures compared to higher temperatures where the WCW decreased (Figure 5B). The results showed that the cultures grew during the induction phase at lower temperatures (10°C) with approximately 2-4% (Table 1). At higher temperatures there was in general a negative change in WCW, indicating cell death. The model showed that a concurrent increase in culture medium pH and decrease in incubation temperature resulted in stronger signals in the ELISA assay as well as an increase in WCW. The yield of TS1-218 increased when the temperature during induction was lowered from 20°C to 15°C and 10°C. These results support the suggestions that lower temperatures decreases cellular death and thus decrease the release of proteases to the culture medium. Although, it cannot be excluded that the lower TS1-218 accumulation at lower pH and higher temperatures could be coupled to other mechanisms in addition to increased protease activity. Different environmental and metabolic conditions during recombinant protein expression induce numeral alterations in host intracellular processes, which are referred to as stress responses [32]. These stress responses can influence the cell metabolism, protein synthesis, folding and secretion mechanism and thus affect productivity of the hosts cells [32]. Gasser et al. [33] have studied the influence of different cultivation temperatures on recombinant protein secretion and transcription of a set of key genes involved in various intracellular processes such as folding, secretion, DNA repair and amino acid synthesis to name a few. They speculated that lower temperatures reduce the amount of stress on the *P. pastoris *folding and secretory machinery enabling a higher production rate of correctly folded recombinant protein. Similar conclusions were made by Dragosits et al. [30] when they studied the effect of temperature on the proteome of *P. pastoris*. In a study by Jahic et al. [34] it was shown that lower temperature gave a 3.5 fold higher AOX activity which together with reduced proteolytic activity resulted in a two-fold increase in recombinant fusion protein production. It should be noted that the *P. pastoris *strains, promoters, culture medium and production techniques in these studies mentioned above differed from the ones employed in our study. It is however, plausible to assume that a combination of altered intracellular processes in the *P. pastoris *cells and a decreased protease release and activity could be responsible for the increased TS1-218 accumulation at higher pH and lower temperatures in our experiments. In our experiments the MeOH concentrations investigated did not have a significant influence on the final yield of TS1-218. This could be explained by the methanol utilization phenotype of our strain (Mut^S^) which utilizes methanol at a much slower rate compared to other methanol utilization phenotypes (i.e Mut^+^).

## Conclusions

The aim of this study was to utilize DoE in order to investigate the effect of pH, temperature and MeOH concentration on production of the anti-keratin 8 single-chain Fv, TS1-218, by *P. pastoris *strain KM71H/Mut^S ^with the purpose to optimize production. The application of DoE facilitated the experimental setup and data analysis and identified several interactions between the investigated factors. The model suggested several factor settings for optimal protein production that deviate considerably from the generally recommended conditions for recombinant protein expression in *P. pastoris*, specially the temperature. The yield of the TS1-218 was improved 21-fold compared to the pre-optimization conditions (pH 6, temperature 20°C, MeOH concentration 0.5%) by lowering the temperature to 11°C and increasing the starting pH and MeOH concentration of the cultures to 7.1 and 1.2% (v/v) respectively during induction. This study shows that *P. pastoris *is highly capable of producing recombinant proteins at temperatures as low as 11°C.

## Authors' contributions

RJ, BS and PH directed and coordinated the study. RJ conducted all experimental procedures, data collection, analysis and interpretation and drafted the manuscript. BS and PH assisted with analysis and interpretation of the model. All authors approved the final version of the manuscript.

## Competing interests

The authors declare that they have no competing interests.
